# Using Machine Learning to Provide Reliable Differentiated Services for IoT in SDN-Like Publish/Subscribe Middleware †

**DOI:** 10.3390/s19061449

**Published:** 2019-03-25

**Authors:** Yulong Shi, Yang Zhang, Hans-Arno Jacobsen, Lulu Tang, Geoffrey Elliott, Guanqun Zhang, Xiwei Chen, Junliang Chen

**Affiliations:** 1 State Key Laboratory of Networking and Switching Technology, Beijing University of Posts and Telecommunications, Beijing 100876, China; 2013213377@bupt.edu.cn (L.T.); xiaozhang123456@bupt.edu.cn (G.Z.); chjl@bupt.edu.cn (J.C.); 2 Middleware Systems Research Group, University of Toronto, Toronto, ON M5S 1A1, Canada; jacobsen@eecg.toronto.edu (H.-A.J.); geoffrey.elliott@mail.utoronto.ca (G.E.); 3 School of Public Health, Indiana University Bloomington, Bloomington, IN 47405, USA; xiwechen@indiana.edu

**Keywords:** software-defined networking, publish/subscribe, quality of service, differentiated service, queue management, machine learning

## Abstract

At present, most publish/subscribe middlewares suppose that there are equal Quality of Service (QoS) requirements for all users. However, in many real-world Internet of Things (IoT) service scenarios, different users may have different delay requirements. How to provide reliable differentiated services has become an urgent problem. The rise of Software-Defined Networking (SDN) provides endless possibilities to improve the QoS of publish/subscribe middlewares due to its greater programmability. We can encode event topics and priorities into flow entries of SDN switches directly to meet customized requirements. In this paper, we first propose an SDN-like publish/subscribe middleware architecture and describe how to use this architecture and priority queues supported by OpenFlow switches to realize differentiated services. Then we present a machine learning method using the eXtreme Gradient Boosting (XGBoost) model to solve the difficult issue of getting the queuing delay of switches accurately. Finally, we propose a reliable differentiated services guarantee mechanism according to the queuing delay and the programmability of SDN to improve QoS, namely, a two-layer queue management mechanism. Experimental evaluations show that the delay predicted by the XGBoost method is closer to the real value; our mechanism can save end-to-end delay, reduce packet loss rate, and allocate bandwidth more reasonably.

## 1. Introduction

In the Internet of Things (IoT) [1,2] scenarios, publish/subscribe (pub/sub) middlewares are used to construct the communication infrastructure for different users to access the massive real-time sensor data. Software-Defined Networking (SDN) [3,4,5,6,7,8] is a promising solution which can be used to solve the hard problem of enhancing the Quality of Service (QoS) about disseminating data over IoT [2]. In the traditional pub/sub middlewares based on IP networks, it will take more latency to match events with the installed filters due to the detour to the broker network. However, in the new pub/sub middlewares based on SDNs, it becomes more efficient to match and forward events directly and fast in SDN-enabled switches by OpenFlow [9] protocol, which standardizes the interface specifications for installing and modifying flow tables directly on SDN switches.

Bakken et al. [10] used the project GridStat to explain the significance of SDN in pub/sub middlewares for IoT services. A pub/sub communication system was deployed over smart grids, subscribers receive events in which they express their interests in advance from the system. To guarantee the QoS about delivering events, they designed some specialized routers. Although the specialized design solved the problem of QoS, it also limited their applications. In software-defined networks, we can avoid the limitation by making full use of the programmability of SDN switches.

Most pub/sub systems consider that all subscribers have the same QoS requirements and make the same contribution to system resources [11]. However, in real-world scenarios, different users may have different delay requirements. Many delay-sensitive and bandwidth-limited IoT applications such as the District Heating Control and Information Service System which has been deployed in Haidian District, Beijing need real-time monitoring, transmission and response to anomalies (e.g., water pipe breakage, fire). We should guarantee that such anomalous or emergency events are processed in real time to prevent any danger. In most existing work about pub/sub middleware [12,13,14,15,16], much more attention has been paid to how to forward events fast and effectively under the assumption that system resources are sufficient, but it is rarely considered how to meet user needs in the case of limited resources. In the user-oriented service scenario, users are often more concerned about whether their needs can be met, such as their end-to-end delay requirements. In this paper, we focus on the problem about meeting more user delay needs under limited network bandwidth. On the other hand, in the traditional network, there is usually only one queue on the output ports of switches because it is difficult to change the factory configuration of switches. We can change this situation by make full use of the programmability of SDN-enabled switches, namely, setting different priority queues on switch outputs. In this way, emergency events are forwarded fast, general events save network bandwidth, and more user needs can be met, realizing differentiated services in IoT environment.

There is some similar work to ours. Tariq et al. [17] proposed a subscriber-driven decentralized method based on the delay demands of subscribers so that subscribers with urgent delay demands are located closer to the related publishers. However, in some location-based IoT applications, this method does not work because subscribers cannot move freely. They also did not take advantage of the flexibility of SDN to set up multiple priority queues for achieving differentiated services. Bhowmik et al. [14,15] presented an SDN-based content-oriented high performance pub/sub middleware PLEROMA, which uses the ternary content-addressable memory (TCAM) of switches to perform filtering operations, realizing the line-rate forwarding of messages, but TCAM is very expensive, power hunger, and has limited storage space. This work focuses on the fast forwarding for all events, but our work mainly considers that emergency events should be forwarded fast, the other events can be processed slowly to free up more bandwidth for emergencies. Wang et al. [18] tried to provide differentiated services by configuring priority queues in SDN switches. However, we found that it was inaccurate to predict the queuing delay of switch ports using the Random Early Detection (RED) method. Because we cannot get the enqueued and dequeued data at the same time, there is often a significant error compared to the real delay. In this paper, we try to solve this hard issue. New methods are proposed to improve the accuracy of predicting the queuing delay; furthermore, we describe a two-layer queue management mechanism based on user requirements to guarantee the QoS of differentiated services. In the end, we also make some comparative experiments about the methods. The goal of this paper is to provide reliable differentiated services in SDN-like publish/subscribe middleware for IoT services.

How to meet the delay requirements of users in IoT is a huge challenge. There are some approaches to achieve it, such as setting message priorities, providing multiple priority queues, configuring system policies, using supervised machine learning methods and providing adaptive transport protocols [19]. In traditional switches, we cannot install and modify flow tables directly on switches. However, SDN switches get rid of these limitations. We can encode event topics and priorities into flow entries of SDN switches directly to meet customized requirements. It is a good way to solve this difficult issue by providing reliable differentiated services based on user delay requirements with priority queues and machine learning methods.

In this paper, we first describe how to provide differentiated services using priority queues for meeting the different delay requirements of users in SDN-like pub/sub systems. Then we propose a machine learning method to solve the difficult issue of getting the queuing delay of switch egress ports, that is to say, using the eXtreme Gradient Boosting (XGBoost) model of machine learning to predict the queuing delay. We can get the queuing delay value by XGBoost accurately; unlike the RED method, there is often a big error compared to the real delay. At last, in order to guarantee the reliability of differentiated services, we use the queuing delay and the programmability of SDN to design a two-layer queue management mechanism based on user requirements. In this way, unreasonable user requirements will not enter the system and bandwidth resources are saved. In particular, to better meet the user requirements, we also design a global QoS control strategy to adjust the delay constraint of each switch dynamically in the whole network. The experimental evaluations demonstrate the effectiveness of our proposed solution.

The major contributions of this paper are as follows:

1. We propose an SDN-like publish/subscribe middleware architecture and describe how to use this architecture and priority queues which we can configure on SDN switches directly to provide differentiated services. The encoding rules about event topics and priorities are presented in detail.
2. To the best of our knowledge, we are the first to predict the queuing delay of switch egress ports using the eXtreme Gradient Boosting (XGBoost) model of machine learning. The difficult problem of how to obtain the queuing delay of switches accurately is solved. We also compare the performance of the XGBoost method with other methods.
3. Based on the above two solutions and making full use of the programmability of SDN, we present a two-layer queue management mechanism based on user requirements to guarantee the reliability of differentiated services from two different perspectives: (1) The local queue bandwidth adjustment algorithm for a single switch in SDN controllers. The bandwidth of each queue of switch egress ports can be readjusted dynamically according to the queuing delay and the queue priority, more bandwidth is saved, and more delay requirements of users are satisfied with limited bandwidth. (2) The global QoS control strategy for all switches on the path from a publisher to a subscriber in the administrator of the pub/sub system. This strategy includes two algorithms: the initial delay constraint calculation algorithm and the dynamic delay constraint calculation algorithm. In this way, we can configure the delay constraint of each switch dynamically and allocate the queue bandwidth more reasonably from the system view, reliable differentiated IoT services are guaranteed.

The remainder of this paper is organized as follows. Section 2 discusses the related work. Section 3 describes the preliminaries about predicting the queuing delay of switches. Section 4 introduces how to provide reliable differentiated services. Section 5 presents the queuing delay prediction method. Section 6 proposes the reliable differentiated services guarantee mechanism. Section 7 provides the experimental evaluations. Section 8 concludes this paper with an outlook on future research.

This paper is an extended version of our paper published in the International Conference on Service-Oriented Computing 2018 [20].

## 2. Related Work

The pub/sub middleware is an event-driven middleware [21]. The full decoupling in time, space and synchronization between publishers and subscribers [22,23] makes pub/sub systems especially appropriate for large-scale distributed IoT service deployments. Pub/sub systems are divided into several variations in the light of different subscription mechanisms, such as topic-based [24,25,26], content-based [27,28,29] and type-based [30,31] pub/sub systems. In the topic-based pub/sub scheme, events are classified by topics, the group communication notion is used. It is easy to implement and does not need too much runtime overhead, especially suitable for real-time IoT service scenarios. However, these systems above are built with overlay networks, their QoS is difficult to improve because clients cannot control the underlying switches. The rise of SDN provides endless possibilities to improve the QoS of pub/sub systems due to greater programmability. The work of [14,15,17,32,33] showed some cases about SDN-based pub/sub systems, they mainly focused on routing and event filtering, the QoS of systems is rarely mentioned.

There have been some works on the QoS solutions of the traditional pub/sub systems. Hoffert et al. [34] used machine learning methods for QoS parameter prediction. For example, neural networks and decision trees are good at protocol classification. We are inspired by these methods to predict the queuing delay of switches using the machine learning model (XGBoost). Zeng et al. [35,36] presented a QoS-aware middleware platform about the composition of web services. A special Service-Oriented Architecture (SOA) infrastructure for monitoring and detection was designed. They tried to maximize user satisfaction for web services composition in middleware, we also use user requirements as the metric of QoS in pub/sub system, but for differentiated services in IoT. Behnel et al. [37] first presented the comprehensive evaluation standard about QoS metrics for pub/sub systems, such as latency, bandwidth, and message priorities. In this paper, we use bandwidth, end-to-end delay, and packet loss rate as evaluation standards. Lu et al. [38] described a new real-time QoS-aware pub/sub service which is in line with the Data Distribution Service (DDS) standard. Wang et al. [39] proposed the first pub/sub message broker which can actively schedule computation resources to satisfy QoS needs. The authors described a message dispatching algorithm to ensure different QoS needs; in our work we propose a two-layer queue management mechanism to improve the QoS of pub/sub system. Pongthawornkamol et al. [40] proposed an analytical QoS model for the timely prediction in distributed content-based pub/sub systems. Basem Almadani [41] presented a QoS-aware real-time pub/sub middleware using DDS to improve the QoS performance in petroleum industry. This article indicates that DDS is a good technology to enhance the performance of IoT service.

However, there are few works about providing differentiated services in SDN-like pub/sub systems. Blake et al. [42] proposed an architecture standard for differentiated services (DiffServ) in Request for Comments (RFC) 2475. The architecture is scalable, a lot of new services can be implemented based on it. The standard also defines the basic architectural model, design principles, multicast considerations, and security considerations of DiffServ. Hakiri et al. [43] in 2013 presented a solution combining differentiated services with DDS to provide the network-level DiffServ in pub/sub systems. Hakiri et al. [44] in 2014 proposed a policy-driven QoS framework that combines Session Initiation Protocol (SIP), DDS, and IP DiffServ to improve QoS in wide-area networks. Aiello et al. [45] compared different queue polices for DiffServ in a single FIFO queue. They supposed that packets are divided into high-priority or low-priority packets, a packet loss strategy and enqueue strategy are designed to implement the DiffServ, the goal is to maximize the sum of the advantages of all packages. but our design aim is to provide the DiffServ in multiple queues of a switch port, different packets have different priorities and enter different queues.

## 3. Preliminaries

In this section, we describe some basic concepts and principles related to define our methods to predict the queuing delay of switches. The queuing delay refers to the time interval from the end of the queue to the start of the queue for a packet. To some extent, the congestion degree of the queue is positively correlated with the queuing delay. The larger the queuing delay, the more congested the queue. Therefore, the queue congestion degree can be judged according to the size of the queuing delay. XGBoost model is a new machine learning model for delay prediction. The Autoregressive Integrated Moving Average (ARIMA) model is used to get the cycle of packets distribution. In this paper, we adopt RED method to predict the queuing delay of switches by RED formula, which is often used to avoid congestion in queue management. The IDM is an improvement of the RED method.

### 3.1. XGBoost Model

XGBoost model is a new and efficient machine learning method proposed by Tianqi Chen in 2016 [46]. This model can solve regression prediction problems in supervised learning efficiently and accurately. It is used very frequently in academia and industry. Compared with the traditional Gradient Boosting Decision Tree (GBDT) algorithm, XGBoost has made a series of improvements in loss function, regularization, and column sampling etc. Whether in the famous machine learning contest Kaggle or KDD Cup, XGBoost is used by many winning teams. It can save system resources and improve training speed which can be 10 times higher than traditional methods.

In [46], the objective function is proposed as shown in Equations (1) and (2). In statistics, Root Mean Squared Error (RMSE) is an evaluation index which is often used to weigh the proximity of the predicted outcomes to the real values [47], so we use the classic RMSE loss function as the evaluation function, as shown in Equation (3).

(1) $$\begin{array}{c} Obj^{\left(t\right)}=\sum_{i=1}^{n}l\left(y_{i},{\hat{y_{i}}}^{\left(t-1\right)}+f_{t}\left(x_{i}\right)\right)+\sum_{k=1}^{K}\Omega \left(f_{k}\right)+const \end{array}$$

(2) $$\begin{array}{c} \Omega \left(f_{k}\right)=\gamma T+\frac{1}{2}\lambda \sum_{j=1}^{T}\omega_{j}^{2} \end{array}$$

(3) $$\begin{array}{c} RMSE=\sqrt{\frac{1}{n}\begin{array}{c} \sum_{i=1}^{n}{\left(\hat{y_{i}}-y_{i}\right)}^{2} \end{array}} \end{array}$$

Here, $l(y_{i},{\hat{y_{i}}}^{\left(t-1\right)}+f_{t}\left(x_{i}\right)$ is the loss function, $\Omega (f_{k})$ is the regularization term. The *l* is a differentiable convex loss function which weighs the deviation between the real value $y_{i}$ and the predicted result $\hat{y_{i}}$, ${\hat{y_{i}}}^{\left(t\right)}$ is the predicted value of the *i*-th instance of the *t*-th iteration, Ω measures the complexity of this model, *T* represents leaves number in the tree. Every $f_{k}$ corresponds to a leaf weight *w* and a separate tree structure, $w_{j}$ is the score on the *j*-th leaf, γ corresponds to the threshold, and the pre-pruning is performed while optimizing to limit the growth of the tree, λ is used to smooth the final learnt weights to prevent overfitting.

### 3.2. ARIMA Model

In statistics and econometrics, and especially in time series analysis, the classic ARIMA model is fitted to time series forecasting. Box and Jenkins first proposed the famous time series forecasting method in 1971 [48]. The ARIMA model has three basic elements expressed as (a,i,m). The autoregressive (AR) element (*a*) indicates the effect of old values on current values in the series; The integrated (*I*) element (*i*) represents that the data values have been taken the place of the deviation between their values and the former values; The moving average (MA) element (*m*) models the model’s random variation as a linear combination of prior error terms [49]. The goal of these elements is to make this model match the data as well as possible.

### 3.3. RED Method

The RED formula [50] is a commonly used algorithm to avoid congestion in packet switching networks. It uses the average queue length to detect the initial congestion. The RED gateway drops packets or sets a flag bit in the header of packets when the average queue length exceeds the default threshold. It enables instantaneous bursts of packets while keeping the average queue length low. The basic principle is that it monitors the average queue length to reflect the queue congestion status. Because of the bursts of the real network load, the queue length varies greatly. The instantaneous full and empty queues cannot be used as a reference for queue congestion. Therefore, the RED formula uses a low-pass filter to smooth transient network changes, focusing on long-term changes in the queue. The RED formula is shown in Equation (4).

(4) avgQ=(1-w)×avgQ+w×qLen

where avgQ is the average queue length, the avgQ on the left in Equation (4) is the current value, and the avgQ on the right is the last one. qLen is the real-time queue length, *w* is the weight, 0<w<1, used to measure the impact of qLen on avgQ. If *w* is close to 0, the change of the real-time queue length qLen will not have too much impact on the average queue length avgQ; If *w* is close to 1, the average queue length avgQ is degraded to the current queue length qLen. Therefore, the choice of *w* is very important. If *w* is too large, the RED formula cannot effectively ignore short-term congestion; If it is too small, then avgQ will react too slowly to the change of the actual queue length. Generally speaking, it is determined by the size and duration of the burst traffic allowed by switches.

The RED method is composed of Equations (4)–(6). This method can reduce the data sensitivity, ignore the instantaneous changes of data, and reduce the resource overhead caused by the frequent bandwidth adjustment. In this paper, the RED formula is different from the RED method. The RED formula is used to get the average queue length avgQ by Equation (4), while the RED method is used to get the queuing delay of switches Delay by Equations (4)–(6).

(5) $$\begin{array}{c} qLen=enQ-deQ \end{array}$$

(6) $$\begin{array}{c} Delay=avgQ/Width \end{array}$$

Here, enQ and deQ are the total number of bytes enqueued and dequeued, respectively. Delay is the queuing delay. Width is the queue bandwidth. We can get enQ and deQ by the Representational State Transfer (REST) API of SDN controllers (OpenDaylight), then we get qLen by Equation (5); Combined with the set *w* value, we can estimate the average queue length avgQ by Equation (4); The queue bandwidth Width is also obtained by the REST API of SDN controllers. At last, we can get the queuing delay of switches through Equation (6).

### 3.4. Incremental Difference Method

This method is an improvement of the RED method. Equation (5) can be improved as follows:

(7) qLen=ΔenQ-ΔdeQ+qLen

where ΔenQ and ΔdeQ are the increment of enQ and deQ, respectively.

Compared with the RED algorithm, this method does not need to guarantee the simultaneity of getting the enqueued and dequeued data, avoiding the influence of measurement time difference on data. At the same time, the error caused by packet loss is no longer cumulative, so the error is greatly reduced.

## 4. How to Provide Reliable Differentiated Services

In this section, we propose some methods used to provide the reliable differentiated services in detail. These methods mainly include four parts. The first part is the architecture design about the SDN-like pub/sub system, it is the system platform to provide differentiated services; The second part is the topic encoding in the topic-based SDN-like pub/sub system, which describes the encoding rules about event topics and priorities; The third part is the design principles of priority queues, which are used to implement differentiated services. The last part is the mechanism used to guarantee the reliability of differentiated services.

### 4.1. SDN-like Pub/Sub System Architecture

**Figure 1:**
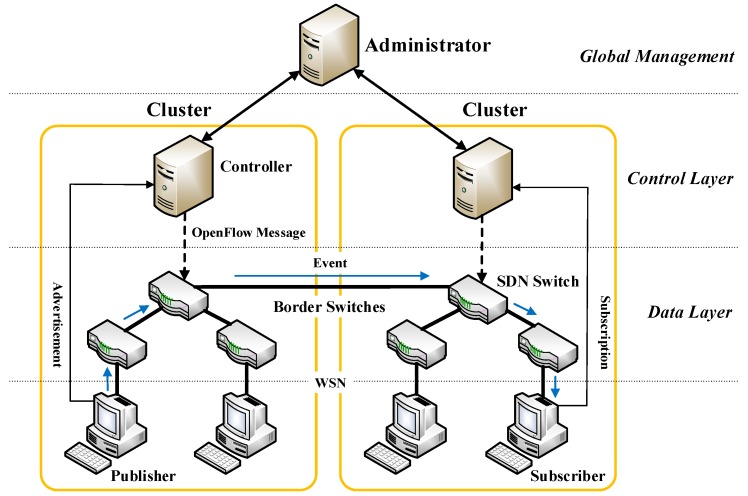
SDN-like pub/sub system architecture.

In SDN, the forwarding plane is separated from the control plane. The control layer is composed of logically centralized controllers, network switches become simple packet forwarding devices. In this way, SDN simplifies the design and management of networks, and makes the network have more programmability and flexibility. We propose an SDN-like pub/sub system architecture, as shown in Figure 1. The system includes one administrator and several clusters. The administrator is responsible for the global network management and interacts with the controller of each cluster. A cluster contains a controller, several switches, publishers, and subscribers. Border switches are used to interconnect clusters. Users communicate with the system by Web Service Notification (WSN).

The system contains three layers: global management layer, control layer, and data layer. Our SDN-like pub/sub system is mainly implemented in the control layer, namely, SDN controllers, which are the control center of the system.

### 4.2. Topic Encoding

**Figure 2:**
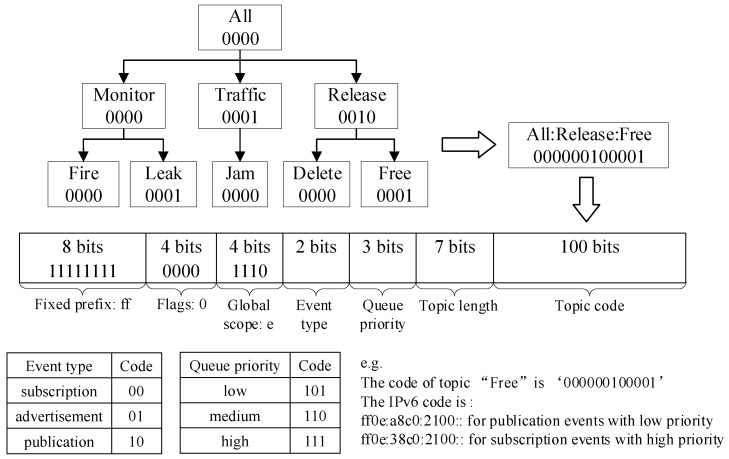
Topic encoding.

In our topic-based pub/sub system, topics are represented as a Lightweight Directory Access Protocol (LDAP) topic tree. The topic, event type and queue priority are encoded into binary strings of the 128 bits IPv6 multicast address in the header of packets. They are used to match flow tables directly when forwarding. If the IPv6 destination address match the flow entries, the events will be forwarded; else they will be discarded. We use 100 bits of IPv6 address to encode the topics. If the number of bits of the topic code is less than 100, we fill them with 0. The encoding rules are shown in Figure 2.

### 4.3. Priority Queue

In this paper, we use different priority queues to meet the different delay needs of subscribers on different topics. Different priority queues have different bandwidths. The bandwidth size determines the forwarding capability of queues. In this way customized differentiation services are provided. In SDN, OpenFlow switches can support up to 8 priority queues per port. These queues are numbered from 0 to 7, and the larger the queue number is, the higher the priority is.

**Figure 3:**
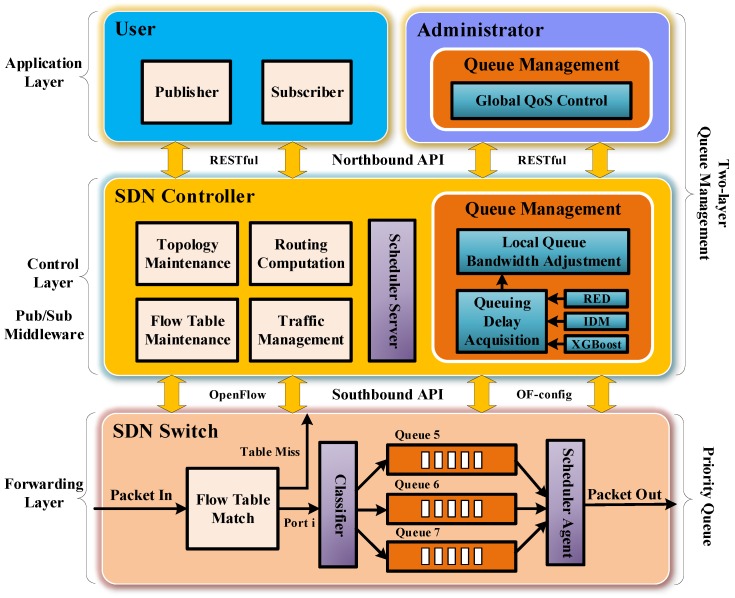
Two-layer queue management mechanism.

For simplicity and ease of experimental verification, we take three priority queues as an example in this paper. In practical applications, we can set more- or less-priority queues according to specific application requirements. In this case, messages are divided into three levels according to their emergency degrees: low, medium, high. The low-priority messages enter queue 5, the medium enter queue 6, and the high enter queue 7, as shown at the bottom of Figure 3. We use 3 bits of IPv6 address to encode the queue priorities of events in case of future expansion, as shown in Figure 2, if the number of levels of the required priority queues is greater than three, we can set more priority queues. When the network is congested, multiple priority queues not only ensure that high-priority messages can be forwarded fast, but also prevent starved problems for low-priority messages.

### 4.4. Reliable Differentiated Services Guarantee Mechanism

The reliable differentiated services guarantee mechanism is a two-layer queue management mechanism based on user requirements. We present the mechanism from two different perspectives, as shown in Figure 3. It is implemented in the control layer and the application layer. Specifically, the local queue bandwidth adjustment is implemented in the SDN controller for a single switch; the global QoS control is implemented by the administrator for all switches on the path from a publisher to a subscriber.

On the SDN controller, we get the queuing delay using three methods: the RED method, IDM, and the XGBoost model, then we use the delay to implement the local queue bandwidth adjustment. The SDN controller communicates with SDN switches using the southbound APIs, such as the OpenFlow protocol [9] and the OF-config protocol [51]. It interacts with the application layer by the northbound APIs, such as REST APIs. The priority queue is mainly implemented in SDN switches.

## 5. Queuing Delay Prediction

The OpenFlow protocol provides standard specifications for interactions between SDN controllers and switches. However, the support for queues is very limited, it does not provide a method to obtain the real-time length of the switch port queue, so we cannot get the queuing delay directly. The RED method [18] uses Equations (4)–(6) to get the queuing delay.

However, For Equation (5), because of the fast changes of SDN networks, it is difficult to get enQ and deQ at the same time by remote access. On the other hand, when the queue is congested, lost packets are counted in enQ but not in deQ, which results in a larger qLen, so the queuing delay is inaccurate. To solve this problem, we propose two other methods. One is IDM as shown in Section 3.4; the other is a machine learning method using the XGBoost model, as shown below.

### 5.1. Data Preprocessing

We collect a large amount of real data, such as bandwidth, package size by capturing data packets from the network and logging once per monitoring period (200 ms). Then we get the queue data after preprocessing by cleaning dummy data, filling missing values, and calculation, some data are shown in Table 1.

The meaning of each field is as follows: ΔTime: the time difference of the packet enqueued and dequeued. ΔStart, ΔEnd: interval with the start or end time. Width: bandwidth. MaxRoute: the maximum number of routing tables. Sum: the total number of received packets. Pkg: package size. ΔIn, ΔOut: the number of in or out of bytes difference from the last packet. ΔInOut: the number of in and out of bytes difference now. Mem: memory size. MaxPkg: maximum packet size. MinTrans: minimum transmission delay. MaxMAC: the maximum number of MAC addresses. The time unit is nanoseconds, rate in Mb/s, and size in bytes.

### 5.2. Feature Selection

#### 5.2.1. Packet Distribution

Packets distribution means the distribution of packet transmission time intervals. The packets distribution shows periodicity, so we use the ARIMA model to obtain the cycle. We also perform a covariance test on the cycle between two adjacent packets. The correlation coefficient is 0.87293211. This higher value shows that there is little difference in their waveform distribution, and this method is reasonable, so we use packets distribution as a feature.

#### 5.2.2. Feature Coding

In raw data, there are many features represented by string that the XGBoost model cannot receive, so we encode them into integer. Data from 11 to 14 columns in Table 1 are encoded as 0, 1, 2, and 4, respectively.

### 5.3. Model Training and Parameter Adjustment

We use the XGBoost model for training. The tree model is easily overfitting, so we divide training set by 20% as validation set and set it as watchlist to obtain the optimal number of iterations. We also set a larger iteration number, and use the score of the verification set no longer declining for 10 generations consecutively as a criterion for early stop.

The RMSE is used as the evaluation function. The smaller it is, the closer the prediction is to the real value. The training results are shown in Table 2. Finally, we choose the first row in Table 2 because it has the smallest RMSE value and can achieve the best results.

## 6. Reliable Differentiated Services Guarantee Mechanism

The reliable differentiated services guarantee mechanism is a two-layer queue management mechanism based on user requirements. User requirements are end-to-end delay requirements of subscribers in pub/sub systems. For a topic, a subscriber can propose a delay as the delay constraint of messages when subscribing. Delay and packet loss rate complement each other: when one declines, the other will rise. In some real-time scenarios, such as live video, the importance of delay is greater than packet loss rate, because the lost data can be retransmitted, but the excessive delay cannot be recovered. Therefore, the goal of system design is to meet delay requirements of users. By this mechanism, more end-to-end delay are saved, we can also adjust the queue bandwidth dynamically based on user requirements to guarantee the reliability of differentiated services.

We achieve our goal from two perspectives. One is the local bandwidth adjustment for a single switch, SDN controllers adjust the bandwidth according to the queue priority and the queuing delay. The other is the global control for all switches on the path. The administrator configures the delay constraint of each switch as the local bandwidth adjustment reference.

### 6.1. Local Queue Bandwidth Adjustment Algorithm

The bandwidth of each queue needs to be readjusted dynamically according to the queuing delay and the queue priority. The higher priority queue should be allocated more bandwidth, and it is not allowed that one queue is congested but another is idle. More bandwidth is saved and more delay requirements of users are satisfied with limited bandwidth. Thus, reliable differentiated services are guaranteed. The constraints for queues are as follows:

(8) $$\begin{array}{c} w_{q}\times t_{q}=AvgQ_{q},q=5,6,7 \end{array}$$

(9) $$\begin{array}{c} t_{q}\leq T_{q},q=5,6,7 \end{array}$$

(10) $$\begin{array}{c} \begin{array}{c} \sum_{q=5}^{7}w_{q}=Port \end{array} \end{array}$$

(11) $$\begin{array}{c} w_{q}>0,q=5,6,7 \end{array}$$

(12) $$\begin{array}{c} Minimize(c_{5}\times t_{5}+c_{6}\times t_{6}+c_{7}\times t_{7}) \end{array}$$

where $w_{q}$ is the bandwidth of queue *q*, $t_{q}$ is the queuing delay, $AvgQ_{q}$ is the average queue length, $T_{q}$ is the delay constraint, Port is the total bandwidth of each switch port. Equation (12) is the adjustment goal, namely, minimizing the weighted delay of queues. $c_{q}$ is queue weight (coefficient), the higher the queue priority is, the greater the value is.

Algorithm 1 depicts the process of the local queue bandwidth adjustment. Here, $t_{q}$ is predicted by the XGBoost model, IDM, or the RED algorithm. Lines 4–6 are determination conditions and updates, we use Equations (10)–(12) to reason and calculate the new bandwidth $B_{q}$, the new delay $D_{q}$. $B_{q}$ is sent to the switch, and $D_{q}$ is fed back to the administrator. The complexity of Algorithm 1 is O(1).

**Algorithm 1** Local Queue Bandwidth Adjustment Algorithm**Input:**$w_{q}$, $t_{q}$, $c_{q}$, $T_{q}$, q=5,6,7 **Output:**$B_{q}$, $D_{q}$1:**Initialize**$B_{q}=w_{q}$, $D_{q}=0$, $c_{q}=q+1$, Port=1002:$AvgQ_{q}=w_{q}\times t_{q}$3:$Sum=\sum_{q=5}^{7}{\left(AvgQ_{q}\times c_{q}\right)}^{\frac{1}{2}}$4:**if**$t_{q}\leq T_{q}$**then**5:    $B_{q}=Port\times {\left(AvgQ_{q}\times c_{q}\right)}^{\frac{1}{2}}/Sum$6:    $D_{q}=Sum\times {\left(AvgQ_{q}/c_{q}\right)}^{\frac{1}{2}}/Port$7:**else**8:    notify the administrator to adjust $T_{q}$9:**end if**

### 6.2. Global QoS Control Strategy

In Equation (9), $T_{q}$ is calculated by the administrator according to user needs and path information. A topic may have many subscribers, we use $U_{j}$ to represent the delay requirements proposed by subscriber *j*. There is a lower delay limit $t_{i}$ for hop (switch) *i*, which is a constant related to switch performance, port bandwidth, and line standards. The switch can guarantee the delay constraint above $t_{i}$. For the whole path, the constraints are as follows:

(13) $$\begin{array}{c} \begin{array}{c} \sum_{i=1}^{n}T_{i}\leq U_{j},j=1,2,...,m \end{array} \end{array}$$

(14) $$\begin{array}{c} T_{i}\geq t_{i},1\leq i\leq n \end{array}$$

(15) $$\begin{array}{c} Minimize(U_{j}-\begin{array}{c} \sum_{i=1}^{n}T_{i} \end{array}) \end{array}$$

Here, *n* is the number of hops, *m* is the number of subscribers of a topic. When paths intersect, a switch may have multiple delay constraints, taking the minimum to meet all needs. We use the best adaptation principle to adjust the bandwidth, that is, to meet user needs as close as possible, as shown in Equation (15). In this way, each user consumes as little bandwidth as possible, more bandwidth is saved; overall, the system can meet more user needs with limited total bandwidth. If it has a solution, the administrator will take $T_{i}$ as the $T_{q}$ of node *i*, and send it to the controller as a local adjustment reference. The *q* depends on the priority of the topic; If it has no solution, the administrator will notify the subscriber by the local controller that the delay requirement cannot be satisfied, please resubmit a new subscription.

After the initial delay constraint is delivered, the administrator still needs to adjust it in real time. SDN controllers periodically feedback the queue delay to the administrator. We use $f_{i}$ to represent the queuing delay fed back by the controller where switch *i* resides, and the administrator recalculates $T_{i}^{\prime }$ of each switch according to the following formulas:

(16) $$\begin{array}{c} Degree=\frac{U_{j}}{\begin{array}{c} \sum_{i=1}^{n}f_{i} \end{array}} \end{array}$$

(17) $$\begin{array}{c} T_{i}^{\prime }=Degree\times T_{i} \end{array}$$

where Degree is the satisfaction degree to which the current delay meets the user needs. $T_{i}$ is the current delay constraint, $T_{i}^{\prime }$ is the new one. In Equations (16) and (17), if Degree>1, it shows that the current delay constraint not only can meet the user needs, but also has some residual delay, $T_{i}^{\prime }$ should become larger than $T_{i}$, so more bandwidth can be saved, the delay requirements of more users can be met with limited bandwidth resources; If Degree<1, it shows that the current delay constraint cannot meet the user needs, $T_{i}^{\prime }$ should become smaller than $T_{i}$; If Degree=1, it shows that the current delay constraint just meets the user needs, no adjustment is required by the local controller. If $T_{i}^{\prime }$ is smaller than $t_{i}$, it shows that the switch cannot meet the current transmission rate, and the administrator should send a notification to the publisher to reduce the frequency of sending packets by the controller. Otherwise, the administrator delivers $T_{i}^{\prime }$ to the controller as the delay constraint of the local bandwidth adjustment. The global QoS control strategy is shown in Algorithm 2 and Algorithm 3.

**Algorithm 2** Initial Delay Constraint Calculation Algorithm**Input:**UserDelay, NodeMinDelay, HopNum **Output:**InitDelayConstraint1:**if**UserDelay<HopNum×NodeMinDelay**then**2:    notify the subscriber to subscribe again3:**else**4:    InitDelayConstraint=UserDelay/HopNum×λ5:**end if**6:save InitDelayConstraint to ConstraintTable7:download the delay constraint message to SDN controller

In Algorithm 2, λ is the allocation ratio, and a flexible space should be reserved for constraints to avoid the extra delay at the sending and receiving end. At last, the initial constraints are saved to the table ConstraintTable, which is used in Algorithm 3. The complexity of Algorithm 2 is O(1).

**Algorithm 3** Dynamic Delay Constraint Calculation Algorithm**Input:**CurrentDelay, LastDelayConstraint, UserDelay, Path, Priority, ConstraintTable **Output:**Res1:**Initialize**Res=0, temp=02:**for**Switch in Path **do**3:    Con=ConstraintTable.get(Switch).get(Priority)4:    temp=temp+Con5:**end for**6:Res=CurrentDelay×UserDelay×temp/LastDelayConstraint

In Algorithm 3, lines 2–5 calculate the sum of the last delay constraint on the path. Line 6 calculates the new constraint and send it to the controller. The complexity of Algorithm 3 is O(n).

This strategy makes full use of the administrator’s characteristics which can control the global network and dynamically allocate constraints based on user requirements. Thus, more reliable differentiated services are guaranteed. In this way, the delay of the entire path can satisfy the user needs, realizing the SDN-like topic-based reliable differentiated IoT services.

## 7. Experimental Evaluation

We perform five experiments to verify the effectiveness of our methods. The first experiment verifies the effectiveness of two new queuing delay acquisition methods and compares them with the RED method. The second and third experiments verify the performance of the local queue bandwidth adjustment algorithm. The fourth one verifies the effectiveness of the global QoS control strategy. The fifth one is a Poisson distribution background traffic experiment to verify the effectiveness of our methods. The last one is constant bit rate and variable bit rate traffic experiments.

In real network scenarios, the queuing delay of switches changes very fast due to the uncontrolled traffic, making it difficult to capture. It is complex and very hard to improve the QoS inside switches, as the queue management inside switches is usually the responsibility of the management software of switches. In this paper, we focus on the queue management at a larger granularity, namely, we explore a two-layer queue management mechanism in the network (across switches), not the queue management inside switches. For network congestion, we focus on the long-term network congestion, not the burst congestion. In this paper, we use the centralized management by SDN controllers and the greater programmability of SDN switches to improve the QoS of SDN-like pub/sub middlewares. SDN controllers can get the topology of the whole network and can know the network status through the northbound API. We can customize the forwarding rules of SDN switches to set queue priorities, differentiated services, and security strategies. Therefore, it is easier to improve QoS in SDN networks.

### 7.1. Experimental Setup

**Figure 4:**
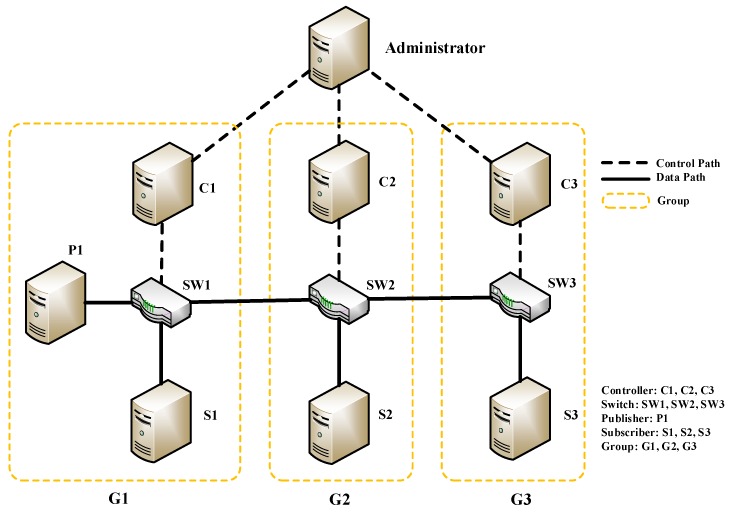
Experiment topology.

We use three SDN-enabled physical switches and several PCs to setup the experiment topology as shown in Figure 4. Each OpenDayLight controller is connected to one or more switches through the OpenFlow protocol. Each SDN controller, switches, and some hosts form a cluster such as G1. The switch model is Pica8-p3290, which can support OpenFlow1.4, Open vSwitch2.3, 48 ports, 8 priority queues, IPv6. The bandwidth of each switch port is 100 Mb/s.

In Figure 4, P1 is a publisher which can send packets with different topics. Different topics have different priorities, and will enter different priority queues. Topics and priorities are encoded into IPv6 multicast address in the header of packets and will match flow tables directly when forwarding. S1, S2, and S3 are three subscribers, each of which can receive packets sent from P1, forming three paths with one, two, and three hops, respectively. For the first three experiments, paths are from P1 to S1 (P1, SW1, S1), For the fourth, the path is from P1 to S3 (P1, SW1, SW2, SW3, S3).

### 7.2. Queuing Delay Prediction Methods Comparison

**Figure 5:**
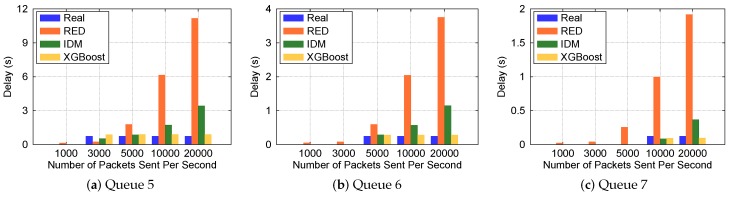
Queuing delay prediction methods comparison.

In this experiment, for simplicity, we assume that the bandwidth proportion of the three priority queues is 1:3:6, so the bandwidths of queue 5, 6, and 7 are 10 Mb/s, 30 Mb/s and 60 Mb/s, respectively. The bandwidth ratio can also be adjusted according to the actual application scenarios. For each queue we run three queuing delay prediction methods under different frequencies of sending packets, the RED method proposed in the work of [18] is the control group of this experiment. The packet size is 1 KB. Packets for each frequency are sent continuously for one minute. The experiment results are shown in Figure 5.

It can be seen from Figure 5 that sometimes the delay of the RED method is far away from the real value, because we cannot get the enqueued and dequeued data at the same time, the value of qLen in Equation (5) is not accurate; However, IDM avoids the measurement time error by Equation (7), therefore, we can get more accurate delay value using IDM by Equation (6). However, according to Equation (4), we cannot get the perfect *w*, the estimated avgQ still has a small amount of error with the real value. The XGBoost method uses a large amount of historical data in the network to predict the delay, it does not need to consider the error of the above two methods, so we can get more accurate delay value than IDM. As shown in Figure 5, when the frequency of sending packets becomes larger (from 1000 to 20,000), the accuracy of IDM becomes worse than XGBoost. In a word, the prediction accuracies of our two new methods are both better than the RED method, and XGBoost is better than IDM, so we choose the XGBoost method for the following experiments.

### 7.3. Local Queue Bandwidth Adjustment Algorithm Verification

**Figure 6:**
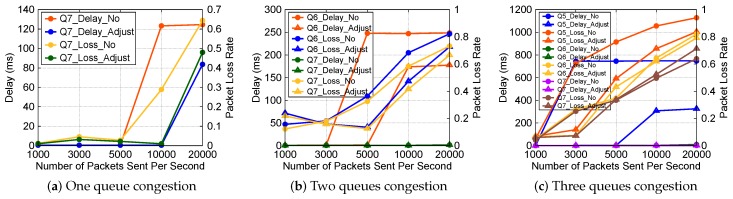
Local queue bandwidth adjustment algorithm verification.

For each combination of three priority queues, we compare the delay and packet loss rate under different frequencies of sending packets. We conduct experiments about one, two, and three queues congestion, respectively. Some experiment results are shown in Figure 6. The initial bandwidths of queue 5, 6, and 7 are still 10 Mb/s, 30 Mb/s and 60 Mb/s, respectively. To prevent starvation of the low-priority queue and avoid less responsive adjustments when data arrive suddenly, we also set bandwidth lower limits to 2 Mb/s, 10 Mb/s and 30 Mb/s for queue 5, 6, and 7, respectively. For *n* queue(s) congestion, there are *n* different topics subscribed, different topics have different priorities.

In Figure 6a, we show the situation about one queue congestion for queue 7. The bandwidth of queue 7 is 60 Mb/s before adjustment, it starts congestion when the frequency is between 5000 and 10,000. The delay remains at 125 milliseconds, and the data received per second also remain in a fixed range, resulting in increasing packets loss. When we use the bandwidth adjustment algorithm, the queue starts becoming congested when the frequency is between 10,000 and 20,000, the delay remains at 83 milliseconds, and the packet loss rate drops significantly compared with no adjustment. At this moment, the bandwidth of this queue is 88 Mb/s, occupying 88% of the whole bandwidth. The data show that this algorithm is effective.

In Figure 6b, we show the situation about two queues congestion for queue 6 and 7. For queue 6, it starts congestion when the frequency is between 3000 and 5000 before adjustment. When we use the bandwidth adjustment algorithm, the queue starts becoming congested when the frequency is between 5000 and 10,000, and the packet loss rate drops significantly compared with no adjustment. For queue 7, there is no significant change in delay, but the packet loss rate is significantly reduced. The data show that this algorithm is effective.

In Figure 6c, we show the situation about three queues congestion for queue 5, 6, and 7. The results are similar with Figure 6a,b. Namely, for each queue, the delay and the packet loss rate decrease significantly compared with no adjustment. The data show that this algorithm is effective.

In Figure 6, when a queue is congested, its delay is always maintained at a stable level if the bandwidth of the queue is not changed, and does not increase with the increase of the packet frequency. In a word, the data show that this algorithm is effective.

### 7.4. Local Queue Bandwidth Adjustment Algorithm Overall Test

**Figure 7:**
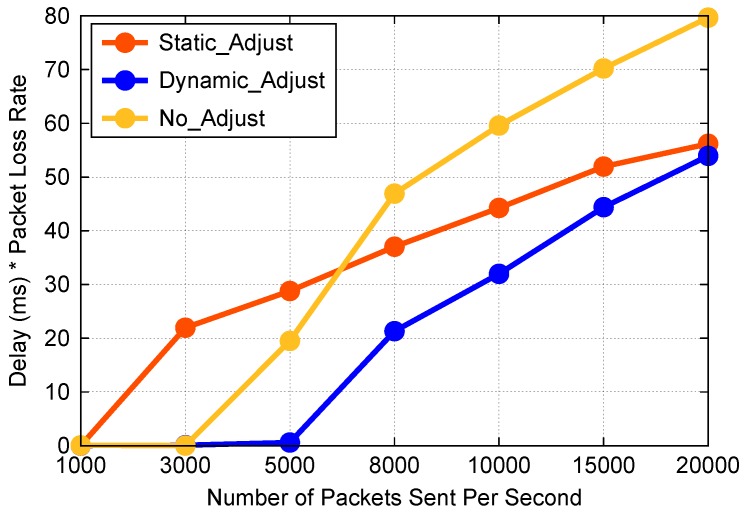
Delay * packet loss rate comparison.

We use the average value of the products of delay and packet loss rate as the metric. There are three settings: the first one has no bandwidth adjustment, the second has static bandwidth, the third adjust the bandwidth dynamically. The static bandwidths of queue 5, 6, and 7 are 10 Mb/s, 30 Mb/s and 60 Mb/s, respectively. The experiment results are shown in Figure 7. The data show that the effect of setting static bandwidth is better than no adjustment and worse than adjusting the bandwidth dynamically, overall. The effectiveness of the algorithm is verified.

### 7.5. Global QoS Control Strategy Verification

In this test, we use the path from P1 to S3, there are three hops (switches) on the path. Each switch has a different delay threshold. We do some experiments on three user end-to-end delay requirements such as 10 ms, 60 ms, 150 ms. Different requirements are assigned to different priority queues, as shown in Table 3.

**Figure 8:**
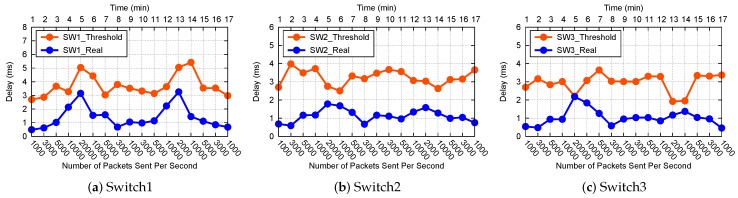
Delay threshold and real delay comparison.

**Figure 9:**
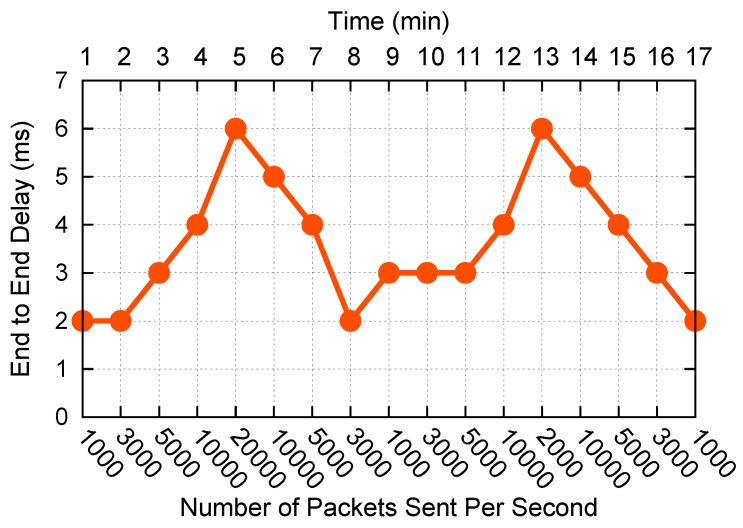
End-to-end delay.

**Figure 10:**
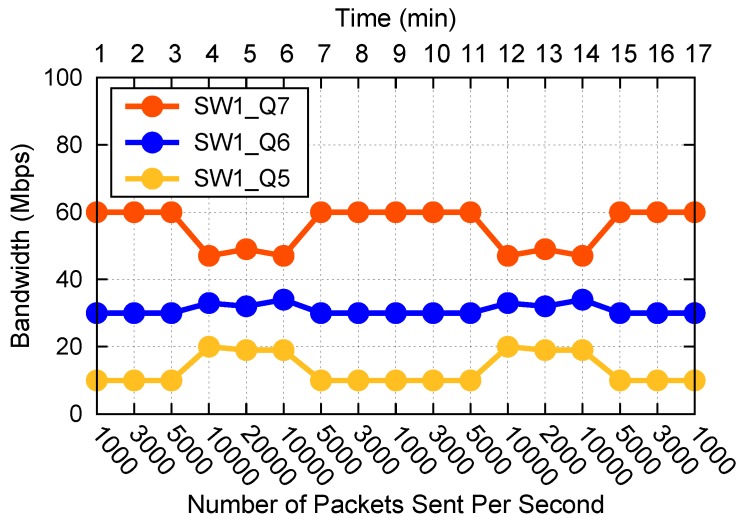
Three priority queues bandwidth.

We show the experiment results about the 10ms user requirements in Figure 8. Figure 8a–c shows that the delay threshold curve is always above the real delay curve for each switch, therefore the threshold settings are reasonable. As shown in Figure 9, the end-to-end delay from P1 to S3 is always less than 7ms, so subscribers can submit their latency requirements which are greater than 7 ms, and the 10 ms user requirements can be met. Figure 10 shows that the bandwidth of the three priority queues of switch1 changes reasonably.

In a word, according to the global QoS control strategy, the administrator can allocate reasonable delay constraints for switches dynamically to meet user requirements. The queue bandwidth of switches can be adjusted dynamically when the network is under the long-term congestion. This experiment verifies that the global QoS control strategy is effective.

### 7.6. Background Traffic Experiment

In the previous four experiments, we verified our queuing delay prediction methods and bandwidth adjustment algorithms with only the application traffic, and no background traffic. In this section, we try to do the experiment alongside a background traffic to simulate the real network scenarios. We adopt a Poisson distribution as the model of background traffic. The Poisson distribution model [52] is one of the most widely used traffic models, which is originally used to analyze traffic in traditional telephone networks [53]. In this model, the number of packets arriving in a fixed time follows the Poisson distribution in the network, and the packet arrival time interval follows the exponential distribution. We can set the time interval of packets arrival as exponential distribution to simulate real network scenarios. The probability function of the Poisson distribution is shown as follows:

(18) $$P\left(X=k\right)=\frac{\lambda^{k}}{k!}e^{-\lambda },k=0,1,2,3,...$$

where λ is the average number of occurrences of random events per unit time. The derivation process is as follows:

The arrival process of data packets in the network satisfies the following three characteristics: 

(I) The arriving packets are independent of each other during different time periods.

(II) In any small period of time Δt, the probability of a packet arrival is independent of the start time, it is only proportional to the length of the time period.

(III) In any small period of time Δt, the number of packet arrivals is either 0 or 1. 

Let β be the probability of a packet arrival in unit time, and let $P_{k}\left(t\right)$ be the probability of *k* packet arrivals in *t* seconds. According to (II) and (III), in any small period of time Δt, the probability of one packet arrival is βΔt, the probability of no arrival is 1-βΔt. If the finite time period *t* is divided into *n* small time periods Δt, namely, t=nΔt, the *k* packet arrivals in *t* can be distributed in any *k*Δt. According to the binomial distribution, the probability of *k* packet arrivals within *t* is as follows:

(19) $$P_{t}\left(X=k\right)=C_{n}^{k}{\left(\beta \Delta t\right)}^{k}{\left(1-\beta \Delta t\right)}^{n-k},k=0,1,2,3,...$$

When n→∞, the binomial distribution follows the Poisson distribution approximately, as shown in Equation (20).

(20) $$P_{t}\left(X=k\right)=\lim_{n\to \infty }C_{n}^{k}{\left(\beta \Delta t\right)}^{k}{\left(1-\beta \Delta t\right)}^{n-k}=\frac{e^{-\beta t}{\left(\beta t\right)}^{k}}{k!}$$

Equation (20) shows that the probability of *k* packet arrivals within time *t* follows the Poisson distribution. $\beta e^{-\beta t}$ is the probability density function of the packet arrival interval, namely, the packet arrival interval is subject to the exponential distribution. Therefore, we can build the Poisson background traffic model: the time interval for each packet sent is an exponential distribution, and the mean of the interval is λ, which is shown in Equation (18).

The Knuth algorithm [54] is used to generate Poisson random variables, and then get the time interval of packets sent by the accumulation of the variables. The Poisson network traffic model parameter value is shown in Table 4.

**Figure 11:**
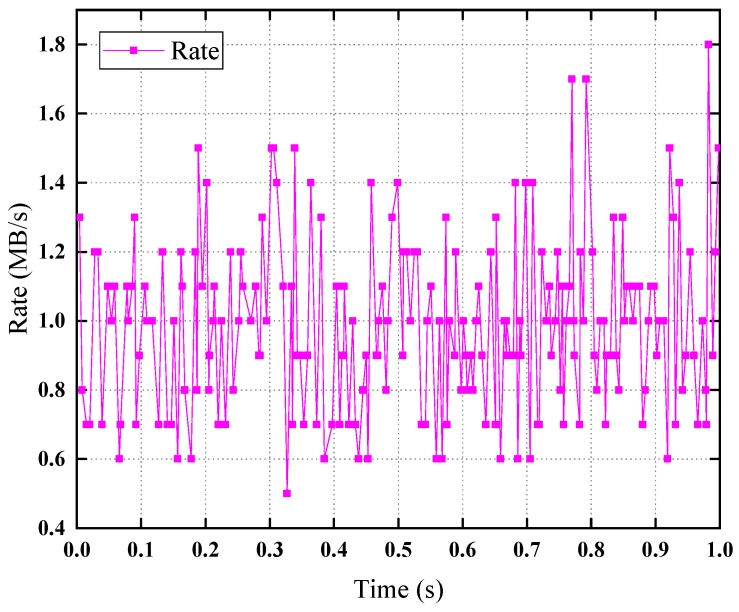
Poisson background traffic model curve.

The Poisson background traffic model curve is shown in Figure 11. In one second, there are about 200 time intervals (points), the intervals follow the exponential distribution, the rate varies around 1 MB/s uniformly, this also meets the characteristics of the Poisson distribution.

**Figure 12:**
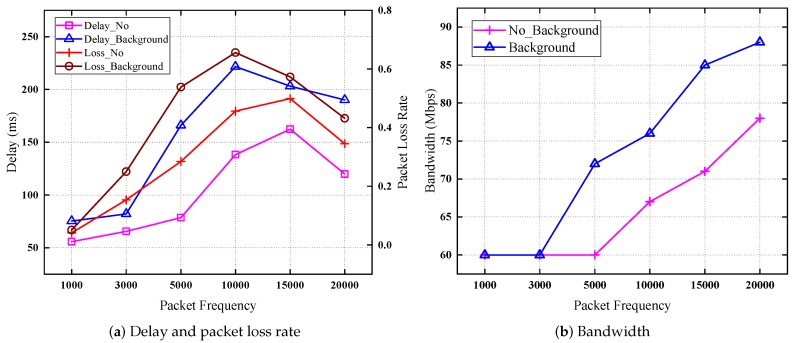
Background traffic and no background traffic comparison.

The comparison of the Poisson background traffic and no background traffic is shown in Figure 12. We conduct two sets of experiments to verify the effectiveness of our two-layer queue management mechanism. One uses the Poisson network traffic model to generate the background traffic, the other one is a control group without the background traffic. The packet size is 1 KB. The packet frequency is the number of packets sent per second. We use different packet frequencies to increase the traffic in the queue. When this value is large enough, the network can generate congestion, then the queue bandwidth adjustment algorithm is triggered to adjust the bandwidth of queues, at last, the delay will become small, and the packet loss rate will decrease. We continue to send each kind of packet for one minute to simulate the long-term network congestion.

We take queue 7 as an example. This queue has the high priority with an initial bandwidth of 60 Mb/s. As shown in Figure 12a, when the background traffic is added to the experiment, the delay increases, and the packet loss rate is also larger than the control group. The experimental group first triggers the bandwidth adjustment algorithm. For example, when the packet frequency is 15,000 pps, the delay begins to decrease for the experimental group, but for the control group, when the value is 20,000, the delay begins to decrease. In Figure 12, the queue bandwidth adjustment algorithm is triggered when the frequency is 5000 for the experimental group, but for the control group, when the value is 10,000, the bandwidth of queue 7 begins to increase. Therefore, our two-layer queue management mechanism is effective, as the bandwidth of queues can be adjusted dynamically under the background traffic.

### 7.7. CBR and VBR Traffic Experiments

In this section, we conduct two traffic experiments with constant bit rate (CBR) and variable bit rate (VBR) to verify our bandwidth adjustment methods. The experiment topology is shown in Figure 4. We choose the path from P1 (publisher) to S1 (subscriber) through only one switch SW1, the experiments are conducted on the Mininet [55] testbed. The Mininet version is 2.3.0, the SDN controller Ryu [56] version is 4.29, the Open vSwitch version is 2.5.0, the OpenFlow protocol version is 1.3, and the operating system (OS) version is Ubuntu 14.04 64-bit.

For traffic generation, we adopt the Distributed Internet Traffic Generator (D-ITG) [57,58], which has been proven to work reliably than other traffic generation tools [59]. It can generate the realistic network workload for emerging networking scenarios. We can get the D-ITG log files after running D-ITG tools. Some average metrics about the network can be dumped as sampled every few milliseconds. In these experiments, we set the time to 1000 milliseconds, namely, one second. Each line of the output file consists of these fields respectively: *Time, Bitrate, Delay, Jitter, and Packet loss.* It is very convenient to evaluate network performance by D-ITG, because we can get experiment results directly without complex calculations. In this paper, we use D-ITG to define two traffic scenarios:

*CBR Traffic.* In this scenario, we generate four flows with constant bit rate in 200 s by D-ITG, respectively. The packet size is 1024 byte. The first flow is used to simulate no congestion in network with the constant packet rate 100,000 packets per second (pkts/s); The second flow is used to simulate the mild congestion with packet rate 200,000 pkts/s; The third flow is used to simulate the severe congestion with packet rate 1,000,000 pkts/s; The fourth flow is used to simulate the severe congestion with the same packet rate after bandwidth adjustments; For the first three experiments, the bandwidth of the switch SW1 is limited to 10 Gbps, for the last one, there is no limit to the bandwidth of the switch, the throughput can be 20 Gbps measured by Netperf [60].

**Figure 13:**
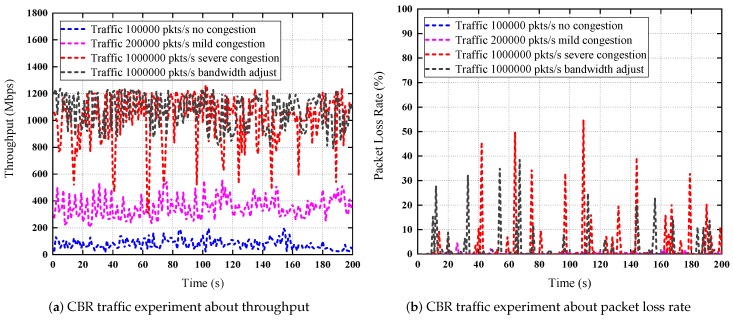
CBR traffic experiments over the Mininet testbed.

The experiment results are shown in Figure 13. Figure 13a presents the traffic from P1 to S1 with different packet rates. Figure 13b presents the packet loss rate about the four flows. We do not use the end-to-end delay as a metric because those delay data are not accurate with virtual machines; therefore, we choose the packet loss rate as the metric for network congestion status, and the throughput is selected as the real traffic metric. For the first flow, there is no packet loss because the packet rate is lower, each packet can be delivered successfully. For the second flow, there is a small amount of packet losses, generally less than 8%, we call the mild congestion for this situation and there is no bandwidth adjustment. For the third flow, sometimes the packet loss rate can be very high, for example, 54.8743% at the 109th second, as shown in Figure 13b, the network congestion is severe, the throughput is low in this second, so the bandwidth adjustment algorithm will be executed in this situation. The execution results are shown in the fourth curve, as shown in Figure 13a, the throughput becomes larger compared with the third flow, the amplitude of the curve decreases, the packet loss rate decreases too. These data prove the effectiveness of our bandwidth adjustment methods. However, the throughout data are not increase too much due to the same packet rate, the real traffic in the network is limited by the packet rate of senders. The throughout data are not stable or not become a straight line under the constant packet rate for each flow because there are bursts and congestions in networks.

*VBR Traffic.* In this scenario, we generate four flows with Pareto distribution for the inter-departure times of packets in 200 s by D-ITG, respectively. The shape parameter λ is 1.75 for all flows, the scale parameters $X_{flow1},X_{flow2},X_{flow3},X_{flow4}$ are 0.01, 0.001, 0.0001, 0.0001 for the four flows, respectively. The four flows are used to simulate no congestion, the mild congestion, the severe congestion, the severe congestion after bandwidth adjustments, respectively. The packet size and the bandwidth upper limit configuration are the same with the CBR traffic experiments.

**Figure 14:**
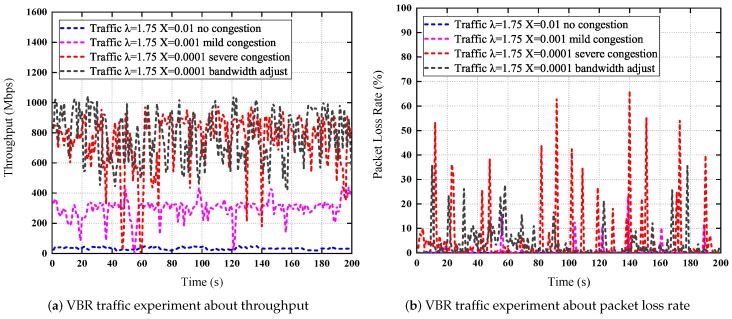
VBR traffic experiments over the Mininet testbed.

The experiment results are shown in Figure 14. Figure 14a presents the traffic from P1 to S1 with different Pareto distribution parameters. Figure 14b presents the packet loss rate about the four flows. For the first flow, there is no packet loss, the throughput is relatively stable. For the second flow, there are some packet losses, the network is in mild congestion, but sometimes the throughput fluctuates considerably, and can be close to 0, this is different from the CBR experiment because the packet rate is changing in Pareto distribution. For the third flow, both the throughput and the packet loss rate fluctuate greatly, the throughput can be 17.54 Mbps, which is close to 0 at the 47th second in the curve, as shown in Figure 14a, and the packet loss rate can be 66.2%, as shown in Figure 14b. These data indicate that the network is seriously congested. After bandwidth adjustments, the throughput becomes relatively stable and larger, and the packet loss decreases too, as shown in the fourth flow in Figure 14. These data verify the effectiveness of our bandwidth adjustment methods.

### 7.8. Discussion

In the queuing delay prediction methods comparison part, one difficult problem for the RED method and IDM is setting the appropriate *w* in Equation (4). If it is too large, the equation cannot effectively filter short-term network congestion, if it is too small, avgQ will react too slowly for changes in the actual queue length. To solve this problem, we select the proper *w* value from many tests according to some network parameters such as the real-time queue length, and the total number of bytes enqueued and dequeued. The other hard issue for XGBoost method is getting enough raw data of switches for training, we collect a lot of real data, such as package size, bandwidth and the total number of received packets by capturing data packets from the network and logging them once per monitoring period (200 ms).

In the local queue bandwidth adjustment algorithm verification part, one difficulty is how to simulate queue congestion. We handle this by increasing the number of packets sent per second gradually. For example, in Figure 6a, when the value of the x-axis is between 1000 and 5000, the queue is not congested, when the value increases to 5000, the queue begins to congest, the delay and the packet loss rate start to increase. The other difficulty is how to reduce the complexity of the local queue bandwidth adjustment algorithm. In the beginning, we use three for-loops to traverse the bandwidth range of each queue, from minimum (the bandwidth lower limits for each queue to prevent starvation) to maximum (the total network bandwidth), but the algorithm complexity is too high, so we use the mathematical reasoning to deduce results directly, reducing the complexity of the algorithm to O(1).

In the local queue bandwidth adjustment algorithm overall test part, the hard problem is how to evaluate the overall performance of the algorithm. Because delay and packet loss rate complement each other, when one declines, the other will rise. For example, if we process packets quickly, the end-to-end delay will decrease, but the packet loss rate will increase due to congestion coming early, so we designed a new metric to reflect system performance comprehensively, namely, the product of delay and packet loss rate. The area formed by the curve and two coordinate axes in Figure 7 reflects the performance of the system to some extent.

In the global QoS control strategy verification part, the difficult issue is how to get the right standard of user delay requirements. We did a lot of experiments to find the right delay boundary. It is not a constant, and varies with the network size, packet size, path length, and so on. As shown in Table 3, we selected three sets of appropriate user delay requirements to prevent the system from returning the resubscription message directly because the requirement could not be satisfied.

In the CBR and VBR traffic experiments, the hard issue is how to simulate the network congestion status. We conducted a lot of experiments and adjusted the parameters for different traffic distribution models. Except the Pareto distribution, there are also Uniform distribution, Normal distribution, and Gamma distribution for the VBR traffic experiments. It is also a meaningful subject to do experiments in real network environments such as campus networks or operator networks.

## 8. Conclusions

In this paper, we mainly address the issue of how to use SDN controllers and SDN switches to provide reliable differentiated services in SDN-like pub/sub middlewares for IoT. In traditional networks, we cannot install and modify flow tables directly on switches; but in SDN networks, SDN switches remove these limitations, we can encode event topics and priorities into flow entries of SDN switches directly. However, existing work about SDN-like pub/sub middlewares rarely studied this issue comprehensively. We first propose an SDN-like pub/sub middleware architecture and describe how to use this architecture and priority queues which we can configure on SDN switches directly to provide differentiated services. Then we present a machine learning method using XGBoost model to solve the difficult issue of getting the queuing delay of switch egress ports accurately. Finally, according to the above two schemes and making full use of the programmability of SDN, we propose a two-layer queue management mechanism based on user requirements to guarantee the reliability of differentiated services from two different perspectives, SDN controllers and the system administrator, respectively. Experimental evaluations show that our solution is effective. In this way, some delay-sensitive IoT services are guaranteed to be prioritized, and the reliable differentiated services are provided to further improve the QoS in SDN-like pub/sub middlewares.

However, we only use three OpenFlow physical switches to do the experiments due to their high costs, we cannot perform large-scale experiments conveniently, therefore it is difficult to involve routing problems. In the future, we hope to do large-scale experiments on a cloud platform with SDN switches or the SDN simulation platform Mininet. In the administrator’s delay threshold settings, it is a new problem to deal with the multi-topic mutual interference. On the other hand, we can combine the local queue bandwidth adjustment algorithm and routing algorithms to promote the QoS of pub/sub middlewares for IoT services.

## Figures and Tables

**Table 1:** Queue data after preprocessing.

Δ *Time*	Δ *Start*	Δ *End*	*Width*	*MaxRoute*	*Sum*	*Pkg*	Δ *In*	Δ *Out*	Δ *InOut*	*Mem*	*MaxPkg*	*MinTran*	*MaxMAC*
2971099	0	0	80	12000	1500000	512	0	0	3351904417	512 M	4 M	1 µs	32 K
2769963	2972088	2770952	80	12000	1500000	512	0	0	3351904417	512 M	4 M	1 µs	32 K
3502000	5742931	6273832	80	12000	1500000	512	0	0	3351904417	512 M	4 M	1 µs	32 K
2793245	9246023	9068169	80	12000	1500000	512	0	0	3351904417	512 M	4 M	1 µs	32 K
2781192	12040209	11850302	80	12000	1500000	512	0	0	3351904417	512 M	4 M	1 µs	32 K

**Table 2:** Training results.

XGBoost Parameter	RMSE
min_child_weight=10; subsample=0.7; colsample_bytree=0.7; scale_pos_weight=0.8; max_depth=4; eta=0.1; early_stopping_rounds=30;	$5.42028\times 10^{7}$
min_child_weight=10; subsample=0.7; colsample_bytree=0.7; scale_pos_weight=0.8; max_depth=6; eta=0.1; early_stopping_rounds=40;	$5.59389\times 10^{7}$
min_child_weight=10; subsample=1; colsample_bytree=1; scale_pos_weight=1; max_depth=10; eta=0.1; early_stopping_rounds=50;	$7.49699\times 10^{7}$

**Table 3:** Delay requirements.

Requirement	Priority	Queue	Bandwidth
10 ms	high	7	60 M/s
60 ms	medium	6	30 M/s
150 ms	low	5	10 M/s

**Table 4:** Poisson background traffic model parameter value.

Parameter Name	Parameter Value
rate	1 MB/s
time interval	5 ms
packet size	1 KB
